# What We Know About TMEM175 in Parkinson's Disease

**DOI:** 10.1111/cns.70195

**Published:** 2025-01-20

**Authors:** Jing Wang, Xuechun Sun, Lufeng Cheng, Meijie Qu, Chanyuan Zhang, Xueting Li, Lingyan Zhou

**Affiliations:** ^1^ Medical Record Information Section Yantai Yuhuangding Hospital Yantai Shandong China; ^2^ Department of Neurology Jinan Central Hospital Affiliated to Shandong First Medical University Jinan Shandong China; ^3^ Department of Neurology Xuanwu Jinan Hospital Jinan Shandong China; ^4^ Department of Neurology Linyi People's Hospital Linyi Shandong China; ^5^ Department of Neurology The Affiliated Hospital of Qingdao University Qingdao China; ^6^ Department of Otolaryngology and Head and Neck Chongqing General Hospital Chongqing China; ^7^ Department of Cardiology, the Second Affiliated Hospital Zhejiang University School of Medicine Zhejiang Hangzhou China; ^8^ Department of Neurology Shandong Provincial Hospital Affiliated to Shandong First Medical University Jinan Shandong China

**Keywords:** lysosome, parkinson's disease, proton channel, TMEM175

## Abstract

**Background:**

Lysosome is a highly heterogeneous membranous organelle in eukaryotic cells, which regulates many physiological processes in the cell. Studies have found that lysosomal dysfunction disrupts cellular homeostasis and is associated with Parkinson's disease (PD). Transmembrane protein 175 (TMEM175) is a lysosomal cation channel whose activity is essential for lysosomal homeostasis. At present, it has been confirmed that TMEM175 is related to the pathogenesis of PD, but the relationship between the two remains unclear.

**Aims:**

A thorough comprehension of the structure and function of TMEM175 would greatly contribute to elucidating the achievement of this objective. In this paper, the structure, composition, and function of TMEM175 and its relationship with PD will be reviewed.

## Graphical abstract

Studies have confirmed that TMEM175 is related to the pathogenesis of Parkinson's disease. TMEM175 may be linked to Parkinson's disease through several mechanisms including α‐synuclein accumulation, oxidative stress, autophagy dysfunction, and neuroinflammation.

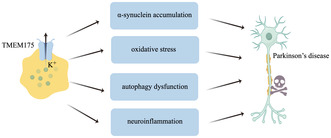

## 1 Introduction

Lysosomes with a single‐layer membrane, known as sac‐like organelles, exist within cells. They contain highly acidic interiors with a pH range of 4.5–5.0 and function as dynamic hubs in the cells [1]. The acidic environment in the lysosomal cavity is the basic condition for lysosomal calcium ion storage, vesicle transport, nutrient sensing, signal transduction, and maintenance of lysosomal integrity, which is crucial for lysosomal function [2, 3, 4]. Lysosomes contain more than 60 acidic hydrolases, such as nucleases, proteases, and lysozymes [5]. The most important function of lysosomes is to use these hydrolases to degrade cellular material from endocytic and autophagy pathways, and to export the generated catabolic products to the cytoplasm for subsequent reuse [2, 6]. In addition, lysosomes can also act as a signaling hub, linking the function of lysosomes to various pathways of intracellular metabolism and nutrient homeostasis by releasing ions and metabolites that have roles such as conducting signals and sensing nutrients [7, 8].

Many types of proteins are embedded in lysosomal membranes, including transporters, pumps, ion channels, and some proteins whose functions are not yet known [9]. Different membrane proteins are involved in the regulation of different lysosomal functions, maintaining normal membrane potential, transmembrane potential gradient, and intracavity ion homeostasis [10]. Under normal physiological conditions, the lysosomal membrane utilizes the vacuolar ATPase (V‐ATPase) as its primary mechanism for creating an acidic environment. By hydrolyzing ATP and pumping H^+^ ions from the cytoplasmic side into the lysosomal lumen, V‐ATPase generates the necessary energy to establish and sustain the low pH within the lysosome [11].

Channels for ions are membrane proteins that form pores, facilitating the passive transfer of ions through the membrane. The lysosomal membrane is abundant in various ion channels, including Ca^2+^, Na^+^, K^+^, and H^+^. The function of lysosomes is dependent on the role of intracavity ions, especially acid‐dependent phagocytosis and autophagy. Ca^2+^, Na^+^, K^+^, and other cations in lysosomes have a crucial function in controlling the electrical charge across the membrane, thereby modulating the influx of protons and the process of making lysosomes acidic. Abnormal lysosome pH can impair lysosome function [6, 7]. At present, reliable proof indicates the crucial involvement of lysosomal dysfunction in neurodegenerative diseases [12, 13, 14, 15]. Abnormal accumulation of protein substances has been found in many neurodegenerative diseases, suggesting that lysosomal degradation may be abnormal. In addition, with the deepening of research, more studies have shown that abnormal lysosomal pH value is involved in the regulation of aging, which is a key inducement factor for a variety of neurodegenerative diseases, restoring lysosomal pH is considered an important strategy for treating these diseases [15, 16, 17]. Disorders affecting the production of energy in mitochondria are also associated with the development of neurodegenerative diseases. Lysosomes play a role in the implementation and control of mitochondrial autophagy, which is an essential process for maintaining mitochondrial integrity. The damaged and aged mitochondria encapsulated by autophagosomes are transported to lysosomes through macrophage, where the mitochondrial autophagy chain is completed under the action of hydrolase, preventing cell damage caused by the accumulation of dysfunctional mitochondria [18, 19]. If the balance of ions in lysosomes is disturbed, the function of hydrolases is compromised and abnormal proteins build up inside lysosomes. This disruption could interfere with the usual breakdown and movement of other lysosomes, ultimately worsening the dysfunction of mitochondria [20]. Therefore, ion homeostasis in lysosomes is critical to lysosome function.

TMEM175 is a porin‐forming protein that is considered to be a K^+^ selective channel and a proton channel on endosomal and lysosomal membranes. Its channel activity holds a vital position in maintaining lysosome and mitochondrial function. Studies have confirmed that TMEM175 deficiency leads to lysosomal pH instability, further reducing lysosomal hydrolase activity, resulting in a decrease in glucocerebroside activity, impaired lysosomal clearance of autophagosomes, and resulting in reduced mitochondrial respiration [21, 22].

PD, the second most prevalent neurodegenerative disease, exhibits a unique amalgamation of motor and non‐motor manifestations. These include bradykinesia, resting tremor, stiffness, postural instability, olfactory impairment, sleep disturbances, autonomic malfunction, and cognitive decline [23]. The underlying cause of PD is the loss of dopamine‐producing neurons and the buildup of α‐synuclein protein. The inflammatory response in the brain, imbalance in mitochondrial and oxidative stress pathways, disruption of autophagy and lysosome processes, and disturbance in synaptic vesicle endocytosis have been implicated in the development of PD [24, 25]. Deficiency in TMEM175 can result in the buildup of neuronal α‐synuclein and the death of dopamine‐producing neurons, which are significant signs of PD [26, 27]. Several recent genome‐wide association studies (GWAS) have discovered a specific gene, TMEM175, as a gene variant linked to a higher risk of PD [28, 29, 30]. The two TMEM175 coding variants with the highest secondary allele frequencies, rs34311866 (p.M393T) and rs34884217 (p.Q65P), were both associated with PD [27, 31, 32, 33]. Thus, the discovery of TMEM175 provides a new therapeutic target and direction for the study of PD.

## 2 Structural Forms of TMEM175

**FIGURE 1:**
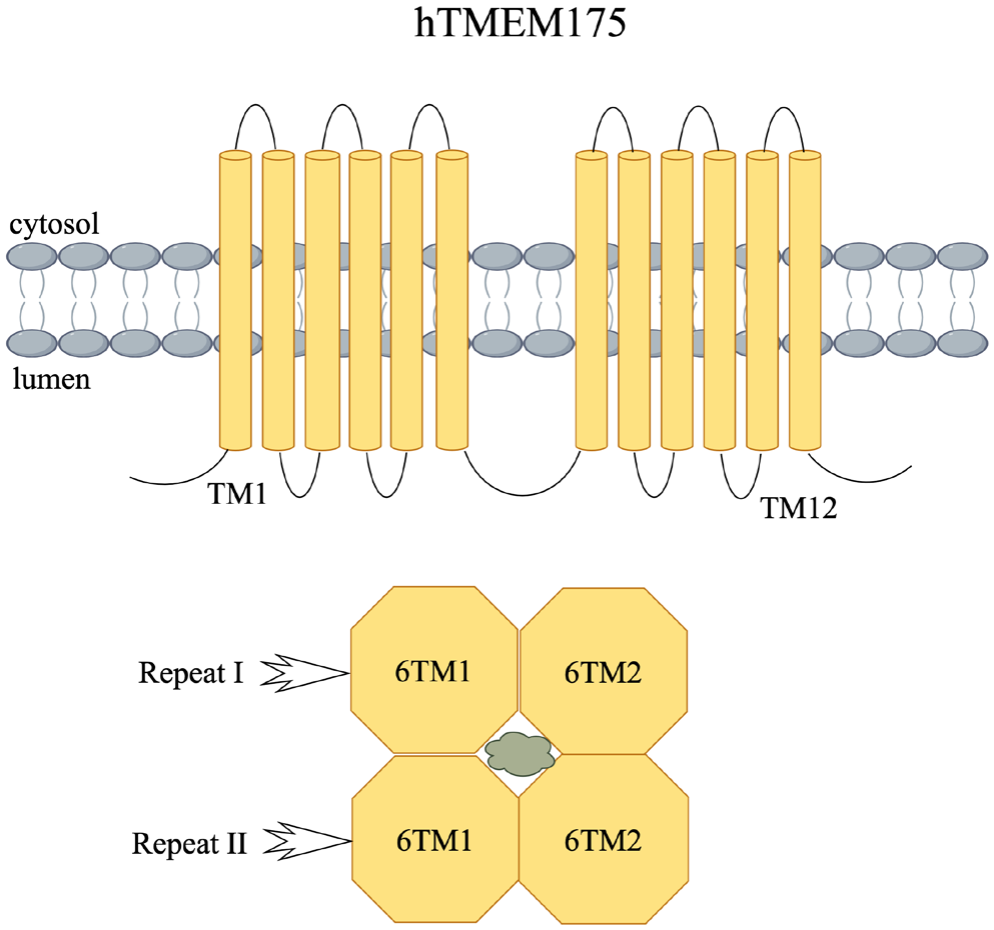
Topological arrangement of hTMEM175 transmembrane monomer (by figdraw).

A protein called TMEM175 serves as a channel that regulates the movement of potassium within lysosomes. It is present in various organisms, including bacteria, archaea, and animals. The molecular structure of human homolog TMEM175 (hTMEM175) is quite different from that of other typical plasma membrane potassium channels (Figure 1). In most potassium ion channels, the high K^+^ selectivity is determined by the structure of the selectivity filter, which consists of a conserved pore (P) ring with a TVGYG‐like characteristic sequence but instead has conserved iso‐amino acid residues as the selectivity filter [27, 34, 35]. Although hTMEM175 is highly selective to potassium, about 20–40 times as selective as Na^+^, it does not contain a P‐ring and instead has conserved iso‐amino acid residues that act as a selective filter. Under low‐temperature electron microscopy, we found the structure of hTMEM175 and its two conformation states: open and closed. hTMEM175 contains two homologous repeat sequences (repeat I and repeat II), each with six transmembrane helices (TM1‐6) that act as dimers in lysosomal membranes [35]. The pathway for ion conduction extends along the axis of homologous repeats located at the center, which is created by TM1 and TM7 in each repeat. In close proximity to the center of the ion conduction hole, a circle of widely conserved isoleucine residues (Ile46 from TM1 and Ile271 from TM7) creates a narrow site of hydrophobic constriction. This leads to the resulting constriction, known as isoleucine‐induced constriction [36, 37]. In the cytoplasm, six transmembrane helices were observed folded and arranged into an hourglass‐like structure, forming a rhombic channel. With dimensions of approximately 85 Å in length and 60 Å in width, the pathway for ion conduction is situated at the core of the channel along the axis resembling four‐fold symmetry. The six spiral structures in each repetition form a separate region, with no visible interchangeable regions or spiral structures. When observed from the plane of the membrane, most hTMEM175 is immersed in the membrane, with small rings protruding on both sides [37].

## 3 The Ion Selectivity and Function of hTMEM175

hTMEM175 was originally described as a lysosomal K^+^ selective channel, and hTMEM175 can permeate Na^+^, Ca^2+^, Rb^+^, Cs^+^, and K^+^. hTMEM175 is more permeable to K^+^ than Na^+^ and Ca^2+^, and its ion selectivity is nearly 40 times that of Na^+^ [32, 38]. The transient dehydration of ions through isoleucine contraction suggests an ion selectivity mechanism. Through isoleucine contraction, the ion channel switches from an open conformation to a closed conformation, preventing the movement of ions coordinated by water molecules, and this contraction is so narrow that, in addition to the help of electrostatic potentials, the ion must shed many water molecules in its hydrated shell to pass through the selective filter. Because the dehydration enthalpy of K^+^ (295 kJ/mol) is lower than that of Na^+^ (365 kJ/mol), K^+^ is more likely to enter a partial dehydration state than Na^+^, and through isocyanine contraction osmosis, can overcome the higher free energy gradient to form a stronger current [36]. This may be the reason why hTMEM175 is highly selective to K^+^ [20, 37]. Although isoleucine constriction is involved in hTMEM175's selective infiltration of ions, it is not clear whether the energy difference between K^+^ and Na^+^ dehydration is sufficient to produce K^+^ nearly 40 times more selective than Na^+^ [21]. Infiltration through selective ion channels results from solvent‐ion interactions, channel‐ion interactions, and, in some cases, interactions between ion‐ions [37]. This interaction is lacking in most of the hTMEM175 pores and is completely absent near isoleucine contraction. Thus, although isoleucine shrinkage is hydrophobic, the unique structure and amino acid composition of hTMEM175 pores facilitate ion penetration. Significantly, the equilibrium of interactions between proteins and solvent ions relies on the ion type. Thus, these opposing forces also regulate the channel's ion selectivity. The ultimate determinant of the specificity of the channel is the combination of the relative expense of dehydrating the ions in competition and the disparity in their interaction energy with the channel [37]. Therefore, it will be important to reveal the roles of TMEM175's distinct selectivity and conduction properties in the lysosome and TMEM175 mutations on the likelihood of PD [39, 40].

As early as 1993, studies have shown that there are proton leakage channels on the endosome and lysosome membranes, which play a key role in the regulation of lysosome pH [41]. In two recent studies, Zheng and Hu et al. [42, 43] found that hTMEM175 is a proton‐selective and proton‐activated channel that mediates the proton leakage current of lysosomes. hTMEM175 has an H^+^‐dependent leakage current when the lysosomal lumen fluid is acidic, and its selectivity and permeability to protons are higher than K^+^. Moreover, the permeability of hTMEM175 to K^+^ was decreased [26, 42]. Furthermore, several studies have also demonstrated that the proton‐donating residues of aspartic acid, glutamic acid, and histidine also contribute to H^+^ permeability. Moreover, the presence of branched‐chain amino acids in ion channels facilitates the transport of hydrogen ions across hTMEM175 [26, 44]. However, the precise mechanisms of strong proton selectivity and whether it also correlates with isoleucine constriction remain to be explored and confirmed. hTMEM175 ion channels are linked to the balance of lysosomes and the onset of PD [22, 29, 45], and further research is needed to determine the factors that control gating and gain insights into the mechanisms by which these stimuli affect the equilibrium between open and closed states, thereby regulating the flux of lysosomal K^+^.

## 4 The Adjustment and Function of hTMEM175

Mitochondria and lysosomes are two key organelles involved in cell metabolism and homeostasis, and the interaction between them plays an important role in regulating the physiological state and function of cells. Maintaining a high acidic lysosomal pH is central to cellular physiology [18]. As a lysosomal K^+^ channel and H^+^ leakage channel, hTMEM175 is involved in the regulation of lysosomal homeostasis, and hTMEM175 has unique pH‐dependent double permeability characteristics [46]. hTMEM175 can selectively conduct K^+^ at neutral pH, but acidic luminal pH transforms it into a highly proton‐selective channel [21, 26, 47].

Lysosomal acidification (proton influx) is achieved by the action of V‐ATPase, which uses the free energy of ATP hydrolysis to pump protons into the lysosome. Counterionic motion is necessary to eliminate the membrane potential created by the proton influx generated by the action of V‐ATPase [47]. The hTMEM175 gene encodes a proton‐activated, proton‐selective channel on the lysosomal membrane (LyPAP), which is required for the lysosomal “H^+^ leak” current [26]. In hTMEM175 knockout (KO) cells, reduced LyPAP currents were found, showing excessive lysosomal acidification and impaired proteolytic degradation [26, 43]. Using electrophysiology, in vivo imaging, single particle cryo‐electron microscopy, and functional proteomics, Zhang et al. revealed that human lysosomal‐associated membrane proteins (LAMP‐1 and LAMP‐2) can directly interact with hTMEM175 and inhibit the activity of hTMEM175 to reduce its proton conduction [48]. Promotes lysosomal acidification to a lower pH environment critical for optimal hydrolase activity. Failure of the LAMP‐hTMEM175 interaction will alkalize the lysosome pH and impair the lysosome hydrolysis function [48]. Studies have shown that hTMEM175 deletion can accelerate the fusion of autophagosome and lysosome in cells, but lead to reduced clearance of autophagy substrates and damage of the autophagy function of lysosome [21, 22, 49].

Normal mitochondria play a vital role in cellular respiration and power generation. By engaging in oxidative phosphorylation within the respiratory chain, mitochondria generate energy crucial for the proper functioning of diverse physiological processes within the cell. Reactive oxygen species is a normal by‐product of cellular aerobic metabolism, which is mainly produced by mitochondria. Jinn et al. [22] found that hTMEM175 defects impaired mitochondrial respiratory capacity, decreased ATP levels, and increased α‐synuclein aggregation. Another study found that in cerebral ischemia–reperfusion‐induced neuronal injury, hTMEM175 overexpression significantly reduces the production of reactive oxygen species (ROS), significantly improves mitochondrial activity, and improves mitochondrial dysfunction [50]. However, Qu et al. [51] found that overexpression of hTMEM175 increased ROS levels, leading to mitochondrial dysfunction. Based on these studies, we boldly speculate that overexpression of hTMEM175 may have a dual effect on mitochondrial function. hTMEM175 promotes mitochondrial activity within a certain range, while excessive hTMEM175 damages mitochondria. Different levels of hTMEM175 loss may lead to different results. Therefore, it may be important to maintain the functionality of hTMEM175 within appropriate limits. However, more evidence is needed to support this hypothesis.

Furthermore, hTMEM175 could also contribute to sophisticated mechanisms of programmed cell death by disrupting the function of mitochondria. The apoptosis regulator B‐cell lymphoma 2 (Bcl‐2), as a key regulator of mitochondrial apoptosis, is mainly localized and applied to mitochondria [51]. Bcl‐2 binds and inhibits hTMEM175 directly and indirectly, and Bcl‐2‐specific inhibitors enhance hTMEM175 activity. Mitochondria are not only the main producers of ROS but also the targets of ROS. A positive feedback loop was found between ROS and hTMEM175, and overexpression of hTMEM175 inhibited mitochondrial autophagy and mitochondrial membrane potential depolarization, leading to mitochondrial damage. Reactive oxygen activation of hTMEM175 may increase the production of reactive oxygen by inhibiting the clearance of damaged mitochondria through mitochondrial autophagy. In contrast, ROS scavengers reduced apoptosis levels and were found to prevent the apoptosis inducer MPP^+^ from activating hTMEM175 [51]. Therefore, hTMEM175 holds great potential to become a promising focus of research in the fields of autophagy, apoptosis, and associated disorders.

## 5 hTMEM175 and PD

PD is a common degenerative disease of the central nervous system that seriously endangers the health of middle‐aged and elderly people. The main pathological features are loss of dopaminergic neurons in the substantia nigra pathway and formation of Lewy bodies, deposition of insoluble α‐synuclein, and other proteins that may lead to cellular toxicity and death [52, 53]. So far, the pathogenesis of PD is not clear. Studies have found that the occurrence and development of PD are closely related to environment and genetics, and the specific mechanisms involve neuroinflammation, mitochondrial dysfunction, oxidative stress, and abnormal protein aggregation [54]. Biochemical and genetic studies have shown that the pathogenesis of PD is related to impaired mitochondrial and lysosome function [55, 56, 57]. hTMEM175, a lysosomal K^+^ channel, has been shown to impair mitochondrial and lysosomal function and increase α‐synuclein aggregation, which lies below the peak of major GWAS for PD, making it a potential candidate risk factor for the disease [22]. Studies have confirmed that hTMEM175 deficiency leads to lysosomal pH instability, resulting in reduced lysosomal catalytic activity, reduced glucocerebrosidase activity, impaired lysosomal clearance of autophagosomes, and reduced mitochondrial respiration [22]. hTMEM175 plays an important role in mitochondrial and lysosomal function and in the pathogenesis of PD and highlights this ion channel as a potential therapeutic target for the treatment of PD [58].

In a study of the pathogenesis of PD in Italian patients, in vitro functional analysis of the ion channel encoded by the mutated hTMEM175 gene showed a loss of K^+^ conductance and a reduced affinity of the channel to protein kinase B (AKT). Impaired autophagy/lysosomal proteolytic flux and increased expression of unfolded protein response markers were also observed in patient‐derived fibroblasts. These data suggested that hTMEM175 gene mutation may be involved in the pathophysiology of PD [59]. Until now, diagnosing PD through genetics has proven to be difficult because of the disease's significant genetic diversity and the challenge in interpreting genetic test outcomes. Gaining insights into the genetic makeup could lead to personalized treatments that address various underlying mechanisms for each person with PD. Two of the most studied variants of the gene encoding hTMEM175 have been linked to PD, with the p.M393T variant with loss of function linked to an increased risk of developing PD, while the p.Q65P variant with a gain of function was the opposite [27, 59] Wie et al. discovered lysoK_GF_, a lysosomal K^+^ channel complex, consists of AKT and TMEM175, and it is activated by growth factors and gated by AKT. Reduction in lysoK_GF_ function predisposes neurons to stress‐induced damage and accelerates the accumulation of pathological α‐synuclein. Their research also revealed the consequences of p.M393T and p.Q65P that are associated with susceptibility to PD [60]. As such, the channel complex may be an attractive target for the development of drugs to alleviate PD.

Increasing evidence revealed that neuroinflammation is an early and critical event in PD pathology [61]. Unregulated or chronic neuroinflammation can contribute to the release of pro‐inflammatory factors that interfere with the repair of neuronal, cause synaptic impairment, mitochondrial dysfunction, loss of neuronal connectivity, and disruption of the blood–brain barrier augmenting the neurodegenerative process [62]. Neuroinflammation is mediated mainly by astrocytes and microglia in the brain. There is substantial evidence that astrocytes and microglia may have a protective or damaging role for neurons, and damaged and overactivated microglia phenotypes are found in the brains of patients with PD [61, 63]. While hTMEM175 is highly expressed in dopaminergic neurons in the dense part of the nigra and microglia in the cerebral cortex, the progression of PD may be driven by a vicious cycle between dying neurons and microglia through oxidative stress, mitochondrial autophagy, and autophagy dysfunction, α‐synuclein accumulation, and pro‐inflammatory cytokine release [64, 65, 66].

The Bcl‐2 has also been shown to be closely related to PD. The imbalance of autophagy and apoptosis caused by changes in the expression and activity of Bcl‐2 may be an important cause of neuronal death in PD patients [67]. Cang et al. [21] described the regulatory roles of hTMEM175 in lysosomal pH, lysosomal membrane potential, and lysosome‐autophagosome fusion. Bcl‐2 binds to and inhibits the activity of hTMEM175, and Bcl‐2‐specific inhibitors enhance hTMEM175 activity, and further inhibit mitochondrial autophagy, disrupt mitochondrial autophagy, and increase the production of ROS. ROS further activates hTMEM175, thus forming a positive feedback loop and promoting apoptosis. hTMEM175 KO has been shown to mitigate motor injury and dopaminergic (DA) neuronal loss in a 1‐methyl‐4‐phenyl‐1,2,3,6‐tetrahydropyridine (MPTP) mouse PD model, induced remarkable neuronal protection [51]. In summary, hTMEM175 can play an important role in the apoptosis signaling pathway and become a potential therapeutic target for PD. The development of specific channel blockers and activators will be an important direction for future research.

## 6 Conclusion

The hTMEM175 has been found to control the electric potential across the lysosomal membrane, maintain pH balance, and facilitate the fusion of cellular structures by regulating the movement of potassium ions on the membranes of endosomes and lysosomes in neurons. The pathological role of hTMEM175 in PD is mainly supported by its regulation of the endocytic and autophagy pathways, as it is the lysosomal potassium and proton channel that regulates lysosomal physiology. The hTMEM175 has also been shown to be involved in apoptosis pathways.

The discovery of hTMEM175 provides new therapeutic targets and new directions for the study of lysosomal‐associated neurological diseases. A study demonstrated that resveratrol restored autophagic flux through the AKT/TMEM175 pathway to attenuate inflammation in rheumatoid arthritis‐associated interstitial lung disease [68]. Another study revealed that knockout of hTMEM175 mitigated motor impairment and dopaminergic neuron loss in a mouse model of PD [51]. 4‐aminopyridine (4‐AP) is a well‐known small‐molecule inhibitor of hTMEM175 [49]. We posit that the structure of the complex with 4‐AP will provide a template for the rational design of such probes, serving as a stepping stone toward deciphering the role of hTMEM175 in lysosomal homeostasis and PD. Currently, the pathogenic mechanism of hTMEM175 is not fully understood, and research on therapeutic interventions targeting hTMEM175 is insufficient. It remains uncertain whether increasing or decreasing hTMEM175 levels could offer therapeutic benefits. Developing specific channel blockers and activators for hTMEM175 will be an important direction for future research. Drugs that effectively control and stimulate the functioning of hTMEM175 may aid in the prevention of neurological disorders.

In conclusion, hTMEM175 appears to be critical to the pathological progression of PD. The studies presented in this review represent many different interactions and pathways, but much is still unknown about the details of hTMEM175 and its role in PD, and more exploration is still needed.

## Author Contributions

L.Z. conceived the project and revised the manuscript. J.W. reviewed the literature and drafted the manuscript. X.S., L.C., M.Q., C.Z., and X.L. helped in the literature search. All authors contributed to the article and approved the submission.

## Ethics Statement

The authors have nothing to report.

## Consent

The authors have nothing to report.

## Conflicts of Interest

The authors declare no conflicts of interest.

## Data Availability

Data sharing is not applicable to this article as no new data were created or analyzed in this study.
